# From genomic to LC-MS/MS evidence: Analysis of PfEMP1 in Benin malaria cases

**DOI:** 10.1371/journal.pone.0218012

**Published:** 2019-06-28

**Authors:** Claire Kamaliddin, David Rombaut, Emilie Guillochon, Jade Royo, Sem Ezinmegnon, Gino Agbota, Stéphanie Huguet, Sayeh Guemouri, Céline Peirera, Romain Coppée, Cédric Broussard, Jules M. Alao, Agnès Aubouy, François Guillonneau, Philippe Deloron, Gwladys I. Bertin

**Affiliations:** 1 UMR 261 – MERIT, IRD, Université de Paris, Paris, France; 2 3p5 Proteomic Facility, Université de Paris, Paris, France; 3 Inovarion, Paris, France; 4 UMR 152 – PHARMADEV, IRD, Paul Sabatier Toulouse III University, Toulouse, France; 5 Centre pour la Recherche et l’Etude du paludisme associé à la grossesse et à l’enfance, Cotonou, Bénin; 6 Institute of Plant Sciences Paris-Saclay (IPS2), CNRS, INRA, Université Paris-Sud, Université d’Evry, Université Paris-Saclay, Gif sur Yvette, France; 7 Institute of Plant Sciences Paris-Saclay (IPS2), CNRS, INRA Université Paris-Diderot, Sorbonne Paris-Cité, Gif sur Yvette, France; 8 CHU-MEL, Pediatric department, Cotonou, Bénin; Ehime Daigaku, JAPAN

## Abstract

**Background:**

PfEMP1 is the major protein from parasitic origin involved in the pathophysiology of severe malaria, and PfEMP1 domain subtypes are associated with the infection outcome. In addition, PfEMP1 variability is endless and current publicly available protein repositories do not reflect the high diversity of the sequences of PfEMP1 proteins. The identification of PfEMP1 protein sequences expressed with samples remains challenging. The aim of our study is to identify the different PfEMP1 proteins variants expressed within patient samples, and therefore identify PfEMP1 proteins domains expressed by patients presenting uncomplicated malaria or severe malaria in malaria endemic setting in Cotonou, Benin.

**Methods:**

We performed a multi-omic approach to decipher PfEMP1 expression at the patient’s level in different clinical settings. Using a combination of whole genome sequencing approach and RNA sequencing, we were able to identify new PfEMP1 sequences and created a new custom protein database. This database was used for protein identification in mass spectrometry analysis.

**Results:**

The differential expression analysis of RNAsequencing data shows an increased expression of the *var* domains transcripts DBLα1.7, DBLα1.1, DBLα2 and DBLβ12 in samples from patients suffering from Cerebral Malaria compared to Uncomplicated Malaria. Our approach allowed us to attribute PfEMP1 sequences to each sample and identify new peptides associated to PfEMP1 proteins in mass spectrometry.

**Conclusion:**

We highlighted the diversity of the PfEMP1 sequences from field sample compared to reference sequences repositories and confirmed the validity of our approach. These findings should contribute to further vaccine development strategies based on PfEMP1 proteins.

## Introduction

Through its asexual development in human erythrocytes, *Plasmodium falciparum* grows and reshapes its host cell. Parasite proteins exported at the host cell surface mediate infected erythrocyte’s adhesion to the host’s endothelium that leads to hypoxia, occlusion and endothelial activation. In cerebral malaria (CM) pathophysiology, the sequestration of infected erythrocytes (iE) in the brain capillaries is believed to trigger coma and brain swelling [1].

Among the proteins exported at the erythrocyte’s surface, the *Plasmodium falciparum* Erythrocyte Membrane Protein 1 (PfEMP1) protein family is involved in cytoadhesion [2]. PfEMP1 proteins are encoded by the multigenic *var* gene family [3–5], consisting in ~ 60 copies per parasite genome [6]. The diversity among *var* sequences is almost endless [7,8] thus participating to the infected erythrocyte ability to evade the immune system. PfEMP1 proteins are high molecular weight transmembrane proteins (200–350 kDa), and are composed of an intra-erythrocytic segment, which is conserved, and a highly variable extracellular segment [9]. The extra-erythrocytic segment is composed of 4 to 9 alternated Duffy Binding Like (DBL) or Cystein Inter Domain Rich (CIDR) domains. The nature and the arrangement of these domains determine the binding phenotype of the iE [9,10]. More specifically, the transcripts coding for the domains cassettes DC8 (DBLα2-CIDRα1.1-DBLβ12) and DC13 (DBLα1.7-CIDRα1.4- DBLβ1/3) are preferentially expressed in severe malaria isolates [11,12].

Among the PfEMP1 receptors in human endothelium, the most common is the broadly expressed in human cell CD36, but PfEMP1 binding to CD36 is not related to any specific form of malaria [13]. Two human host receptors for PfEMP1 binding in the context of severe malaria have been identified: the InterCellular Adhesion Molecule-1 receptor (ICAM-1) [14] and the Endothelial Protein C Receptor (EPCR) [15], both expressed in brain endothelial cells [16], and co-localized with the sequestered iEs in severe malaria [14]. The binding domain for ICAM-1 receptor is located in the C-terminal third of the DBLβ3 [17], and the residues involved in PfEMP1 binding to ICAM-1 are highly variable with a limited binding pattern [18,19]. The role of EPCR in PfEMP1 binding has been more recently shown [15] and is still an important research problematic [20]. EPCR binding is mediated by highly variable but structurally conserved CIDRα1 PfEMP1 domains (more precisely CIDRα1.1 and CIDRα1.4–1.8) [21,22]. Importantly, the level of PfEMP1 transcript associated with EPCR binding is higher in samples from patients suffering from severe malaria and increases with the severity of the disease [20,21]. A dual binding with EPCR and ICAM-1 has been suggested, since not all CM isolates present an increase in binding-EPCR PfEMP1 coding transcript [23]. The expression of DBL involved in ICAM-1 binding is associated with dual ICAM-1 and EPCR binding [19].

Most field studies looking for *P*. *falciparum* binding phenotypes are based on molecular biology analysis and have shown that transcript coding for specific PfEMP1 domains expression level is associated with disease outcome [11,21,24,25]. However, this strategy is currently limited to the already identified PfEMP1 domains and does not give proficiency of the expressed proteins. Recently, several strategies have been implemented to investigate the variability of full-length *var* genes using whole genome sequencing [6,26,27] or dedicated long range sequencing with a hybrid PCR approach [23]. In addition, Tonkin Hill et *al* performed *de novo* assembly of *var* genes issued from RNA sequencing (RNAseq) and identified transcripts up-regulated in severe malaria [28]. These recent publications provide insight towards *var* genes variability in the studied areas. However, the identification of PfEMP1 proteins by mass spectrometry approach (LC-MS/MS) remains infrequent in publications.

To complement this deficiency, we aimed to conduct a mass-spectrometry-based proteomics analysis of *P*. *falciparum* field isolates proteome. LC-MS/MS is a powerful and sensitive tool for protein identification, however, its application for PfEMP1 identification remains challenging because PfEMP1 has highly variable sequences, yet database repository is usually simplified by eliminating redundancy. That is the reason why they do not reflect the natural sequence diversity that may occur in such a context.

**Fig 1:**
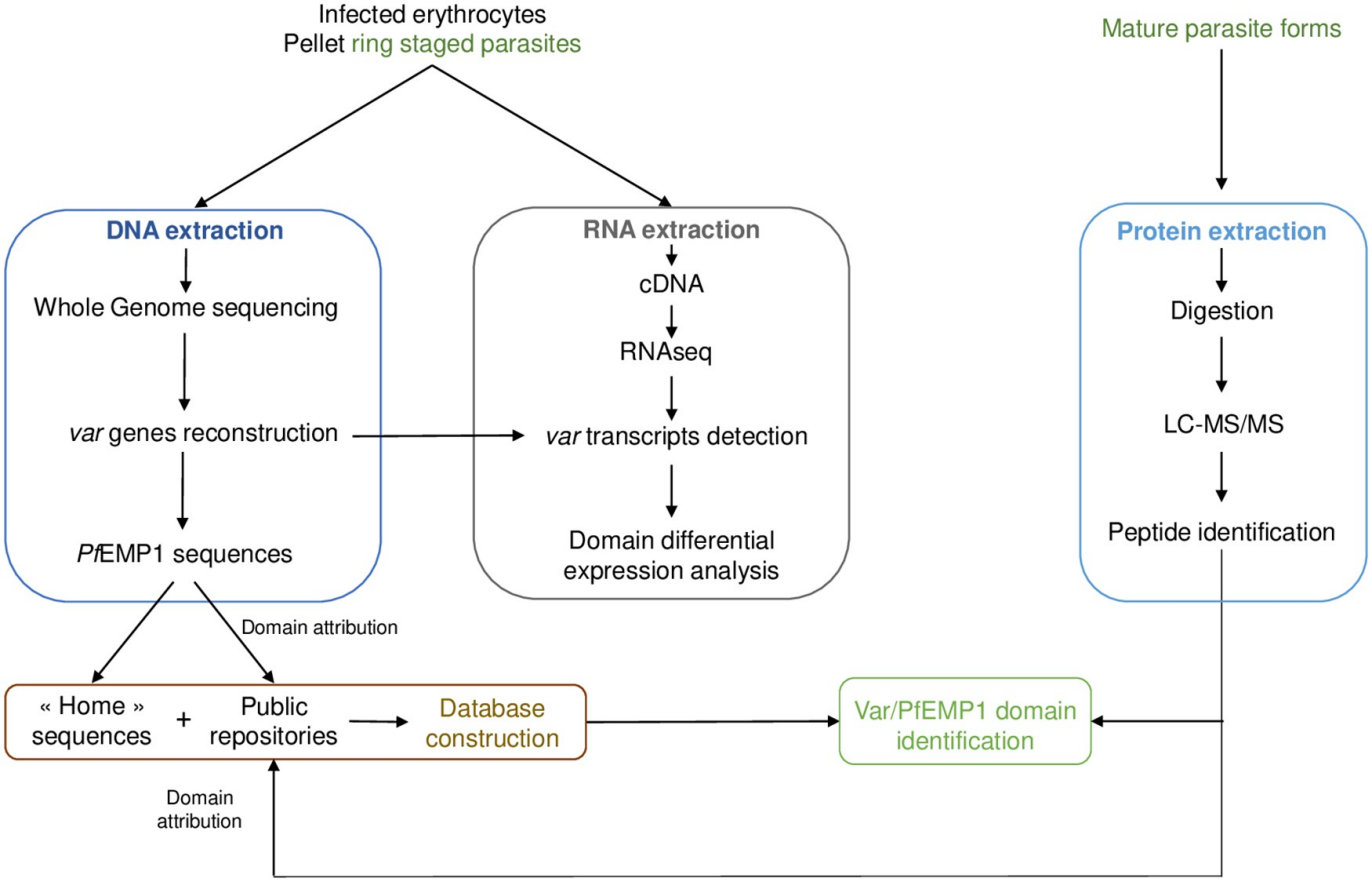
Experimental design—Proteogenomic approach on field samples for PfEMP1 identification. Whole blood sample from patients are collected. DNA and RNA are extracted from parasite’s ring forms. For LC-MS/MS analysis, parasites are matured, and the corresponding proteins are extracted and analysed using the mass spectrometer. Whole genome sequencing data provides the var repertoire from each isolate and allows the assessment of RNA expression in each sample. In addition, WGS data were used to enrich the protein database for protein identification with LC-MS/MS data.

To identify PfEMP1 associated with *P*. *falciparum* clinical outcome in endemic settings, we used a “proteogenomic” approach. Specific PfEMP1 sequences from each isolate were reconstructed *de novo* using whole genome sequencing (WGS) data to identify the expressed transcripts and enrich the protein database (Fig 1). We analysed the whole proteome of samples from patients presenting CM, Severe Anemia (SA) or Uncomplicated Malaria (UM), and attribute PfEMP1 sequences within these samples. Corresponding samples were analysed in RNAseq for PfEMP1 expression analysis, in relation to proteomic results.

We performed RNAseq successfully on 7 field samples (3 CM, 2 SA and 2 UM) and managed to identify the PfEMP1 protein sequence associated with 4 CM samples, 9 SA and 9 UM samples from Benin, West Africa, using LC-MS/MS. We confirmed the expression of several PfEMP1 within a single field isolates and provided the first identification at the patient’s level of PfEMP1 expressed by the parasite in the context of acute *P*. *falciparum* infection (Fig 1).

## Material and methods

### Ethic statement

Ethical clearance was obtained from the Institutional Ethics Committee of the faculty of health science at the Abomey-Calavi University in Benin (clearance n°90, 06/06/2016). Before inclusion, written informed consent was obtained from children’s guardians. Patients were treated in accordance to the national malaria program policy. The methods were carried out in accordance with the relevant guidelines and regulations.

### Sample collection

Patients under age of five, presenting *P*. *falciparum* acute infection, were included in the Lagune Mother and Child Hospital in Cotonou (severe malaria), Benin and Saint-Joseph Hospital, in Sô-Ava, Benin (UM) in rainy season (May–August) 2016. Severe malaria patients were classified as following: CM was defined as associated with a coma (Blantyre score ≤ 2) and the absence of meningitis detected by CSF count and culture and SA was defined with Hb < 5g/dL, measured using Hemocue device (Radiometer). UM was defined as a *P*. *falciparum* infection with fever, in the absence of any other complication. Five mL of peripheral whole blood were collected on EDTA. Parasite density was evaluated with Giemsa-stained thick blood smear. Only pure *P*. *falciparum* infections were retained for the study. Samples were depleted from white blood cells using a gradient-based separation technique Ficoll (GE Healthcare Life Science).

### Whole genome sequencing

Fifty μL of erythrocyte’s pellet was extracted using DNEasy Blood kit (Qiagen). WGS was performed by the Malaria Genomic Epidemiology Network (MalariaGEN) at the Welcome Trust Sanger Institute (Hinxton, UK). Reconstructed *var* genes were kindly provided by Thomas Otto, Matt Berriman and Chris Newbold from the Welcome Trust Sanger Institute and translated into putative PfEMP1 protein sequences for protein identification. The raw reads from whole genome sequencing are available on the ENA server under the accession number listed in S1 Table.

### Transcriptome studies of ring staged parasites

Ring staged parasites were preserved in 5 volumes of pre-warmed (37°C) TriZol (Life Technology), vortexed then immediately frozen at -80°C until further utilization. RNA were extracted as described [29], then digested with DNAse I (Qiagen) and purified using RNEasy MinElute Cleanup kit column (Qiagen). Only RNA presenting a RNA Integrity Number (RIN) > 7 evaluated with PicoChip Agilent 2100TM Bioanalyzeur (Agilent) were retained for downstream analysis [30]. RNAseq libraries were performed using TruSeq Stranded mRNA protocol (Illumina, California, U.S.A.). RNAseq samples have been sequenced in paired-end (PE) with a sizing of 260 base pairs and a read length of 150 bases. Fifty four samples by lane of Illumina NextSeq500 (IPS2 POPS platform) were generated using individual barcoded adapters. Approximately 5 million of PE reads by sample were obtained. The raw reads (fastq) were trimmed with Trimmomatic [31] tool for Phred Quality Score Qscore >20, read length >30 bases, and ribosome sequences were removed using sortMeRNA [32]. RNAseq paired-end reads were mapped to the human reference genome Hg38 (UCSC Genome Browser). Unattributed reads were mapped to the *P*. *falciparum* 3D7 strain reference genome (PlasmoDB release 41), the reference *var* genes removed and replaced by the *var* genes of each sample issued from its own whole genome using HISAT2 (v2.1.0) [33].

Raw counts for each *var* transcript were obtained using HTSeq-count (0.11.1) [34]. Transcript abundance was evaluated using RPKM values. We considered a transcript as present if the RPKM value was > 1. To assess the potential expression differences according to the sample group (patients’ clinical presentation—severe or uncomplicated malaria), we performed a selective read count on each *var* domain subtype from the cognate isolate *var* transcripts. The differential expression analysis was performed on the obtained counts using DESeq2 R package [35].

### Proteome analysis of *P*. *falciparum* late trophozoites using LC-MS/MS

Blood samples were matured *in vitro* for 18 to 32 hours in RPMI medium supplemented with human serum and Albumax (Gibco) and preserved after MACS (Myltenyi Biotech) enrichment as described [36].

Whole cell infected erythrocyte lysates were solubilized and digested in solution using trypsin (Promega, sequencing Grade). Briefly, 50 μg of proteins from whole cell lysates were diluted to 25 μl in solubilization buffer (1% sodium desoxycholate, 100 mM Tris/HCl, pH 8.5, 10mM TCEP, 40 mM chloroacetamide), heated for 5 min at 95°C and sonicated three times for 30 s. Once at room temperature, extracts were diluted with 25 μl Tris-ACN buffer (50 mM Tris/HCl pH 8.5, 10% ACN). Collected peptides were fractionated in 5 fractions per sample by strong cationic exchange (SCX) StageTips [37].

LC-MS/MS analysis was performed on a Dionex U3000 RSLC nano-LC-system coupled to an Orbitrap-fusion mass spectrometer (Thermo Fisher Scientific) as described [38]. Peptides from each SCX fraction were solubilized in 0.1% trifluoracetic acid (TFA) containing 10% acetonitrile (ACN) and were separated on a C18 reverse-phase resin (75-μm inner diameter and 15-cm length) with a 3-hr gradient. The mass spectrometer acquired data throughout the elution process and operated in a data-dependent scheme.

For protein identification using LC-MS/MS, we created a custom database containing both the human proteome (to identify peptides issued from the erythrocyte) and *P*. *falciparum* proteome. In order to perform PfEMP1 protein identification, we concatenated *P*. *falciparum* proteins sequences from PlasmoDB (v35), Uniprot and NCBI. In addition, we implemented our own PfEMP1 sequences, obtained after *in silico* translation from *var* genes reconstruction. Duplicate sequences were removed.

The LC-MS/MS data were analyzed using MaxQuant version 1.5.2.8 [36] as described [39]. The database used was our homemade database and the list of contaminant sequences from Maxquant. For analysis, LFQ results from MaxQuant were imported into the Perseus software (version 1.5.1.6). Reverse and contaminant proteins were excluded. Only proteins from *P*. *falciparum* were selected for further analysis. We then focused on the membrane associated and putative proteins from *P*. *falciparum*.

### Analysis of *var* transcripts and PfEMP1 proteins

PfEMP1 sequences from expressed *var* transcripts and proteins identified in LC-MS/MS were aligned using the VarDom server against reference sequences for domain identification [7]. We specifically searched the pattern for ICAM-1 binding I[V/L]x_9_N[E]GG[P/A]xYx_27_GPPx_3_H [19] using the ProSite online interface [40]. To identify the nature of each domain within the identified sequences from RNAseq and LC-MS/MS, we aligned each DBL and CIDR domain with the VarDom database domain sequences using MAFFT tool (v7) [41]. Using the MAFFT output, we generated a phylogenic tree using PhyML online tool with default parameters [42]. Results were displayed using iTOL online tool [43]. PfEMP1 domains were attributed to all identified peptides. We considered a peptide specific of a subdomain if a peptide was corresponding to the same subdomain in at least 3 different PfEMP1 proteins.

### Statistical analysis

Patient’s samples information’s were compared between the 3 patient’s groups (UM, CM and SA) using one-way ANOVA. Bonferroni’s Multiple Comparison Test was applied for individual group comparison. We considered a *p* value < 0.05 as significant. Qualitative data were compared with Chi Squared test using contingency table. All analyses were performed using Prism v5 (Graphpad). For the differential expression analysis, a domain subtype was considered as differentially expressed in a condition compared to another for log2 (fold-change) value > 1 and adjusted p-value < 0.1.

## Results

### Included samples

We included 95 patients, covering 31 SA, 18 CM and 46 UM. The average patient age was similar among all inclusion groups. Parasite density geometric mean was 8,055 p/μL for UM group, 34,191 p/μL for CM and 24,313 p/μL for SA. Parasite density was only significantly different between SA and UM samples (*p* = 0.0158 with Bonferroni’s Multiple Comparison Test). Hemoglobin level was measured for 20 UM, 17 CM and 31 SA, respectively 11.28 [10.26; 12.75], 5.51 [4.10; 6.56] and 4.393 [3.90; 5.00] g/dL. Hemoglobin level was statistically different for UM samples (*vs*. CM and *vs*. SA) (*p* < 0.05). No difference in erythrocyte count (*p* = 0.1274) and temperature (*p* = 0.9125) was retrieved between SA and CM. All CM patients presented a coma (average BS 2 [2; 2]), while SA patients did not (average BS 4.6 [4; 5]) (*p* < 0.0001).

For LC-MS/MS analysis, we selected samples among those which showed successful maturation. The analysis has been performed on 4 CM, 9 SA and 9 UM samples. 25 samples qualified for RNAseq among which 7 were successfully sequenced.

### *Var* genes transcripts identification with RNAseq

Overall 165 *var* transcripts were identified (S2 and S3 Tables) among which 134 sequences corresponded to Severe Malaria (SM) samples (52 CM and 82 SA) and 31 to UM samples. We then focused on the corresponding sequences domains combination, considering the sequences with at least one NTS domain. We found 102/134 *var* transcripts in the SM groups (30 in the CM samples and 72 in the SA samples) and 24/31 of UM associated *var* sequences.

Among these sequences, the domain combination the most representative is NTS-DBLα-CIDRα-DBLδ. This combination was identified in 17/24 (71%) UM samples and in 45/102 (44%) SM group thus 10/30 CM and 35/72 SA associated *var* transcripts.

The second domain combination is NTS-DBLα-CIDRα-DBLβ and corresponded to 6/24 (25%) of UM associated *var* transcripts and 42/102 (41%) of SM (17/30 CM and 25/72 SA) associated *var* transcripts.

Regarding the domain cassette distribution, we have identified 2 DC8 but no DC13 among *var* transcripts of CM samples, and 2 DC8 and 3 DC13 were identified in SA samples. From to UM samples, we have identified neither domain cassettes DC8 nor DC13.

The specific search of the binding pattern for ICAM-1 retrieved three identifications from CM samples, two in the SA group within the *var* transcripts sequences and no identification among the UM samples.

**Fig 2:**
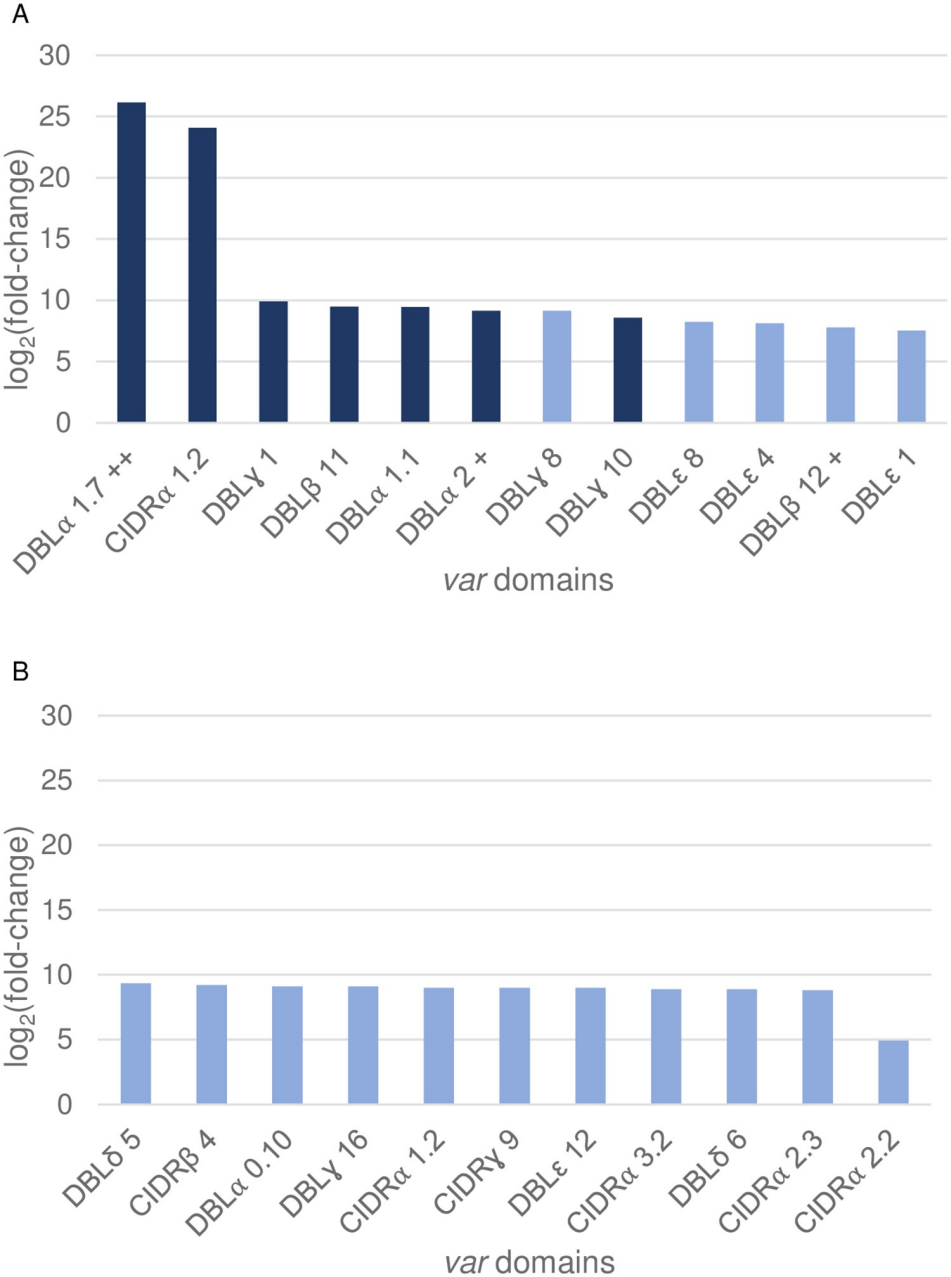
Var transcripts associated domains subtypes identified as up regulated. The bar graph represents the expression differential of the sub-domains realized with the package R DESeq2 (A) overexpressed in the CM in comparison to UM samples and (B) overexpressed in the SA in comparison to UM samples. The signs + and ++ represents respectively the subdomains of DC8 and DC13. Y axis plots the values of log2 (fold change) between the clinic groups by subdomain. X axis represents each domains subtype identified as up-regulated in clinic groups Two adjusted p-value thresholds are indicated: dark blue < 0.05 and light blue < 0.1.

In addition, we performed a differential expression analysis on the *var* domains subtypes of each sample. Twelve domains subtypes were up-regulated in CM samples compared to the UM samples (Fig 2A), among which the DBLα2 and DBLβ12. These domains match to the organisation of DC8. The DBLα1.7 domain (part of DC13) is the most differentially expressed in the CM samples compared to the UM samples.

Eleven domains subtypes were up-regulated in SA compared to UM (Fig 2B). These subtypes were different from those found in CM compared to UM and not correspond to domain cassettes. We found no significantly expressed domains subtype in the CM samples in comparison to SA samples.

### Protein identification using LC-MS/MS

Protein identification was performed using a homemade database (reference sequences from human and *P*. *falciparum* repositories, and the assembled *var* from field samples) containing 295,601 protein sequences, among which 87,489 were *P*. *falciparum-*associated sequences. Overall, we identified 3300 proteins. A total of 1302 proteins were associated to the human proteome, and 1912 to *P*. *falciparum’*s. Among those later, 460/1912 proteins were identified as *P*. *falciparum* membrane-associated proteins, including 60.4% of hypothetical or putative, 12% of PfEMP1s, 3.5% of RIFINs, 0.9% of STEVORs, 1.5% of PHISTs and 21.7% belong to other protein families.

A total of 57 proteins associated with PfEMP1 were identified. Only 10 of the identified PfEMP1 using LC-MS/MS (as part of the identified isoforms) were known sequences from public database repository (Uniprot and PlasmoDB). All other identified PfEMP1 sequences resulted from the translation of the reconstructed *var* genes from our samples (S3 Table).

### PfEMP1 identification and composition

Average molecular weight of the identified PfEMP1 was 228.3 kDa. Using the VarDom online server, we reconstructed the domain architecture from the identified proteins (S4 Table) and we were able to identify domains in 54 out of 57 sequences. NTS domain was found in 41/54 of the sequences (76%) and 39/41 (95%) of the sequences identified presenting an NTS domain displayed DBLα-CIDRα associated to the NTS domain. The three-major head-terminal domain organizations were the following: NTS-DBLα-CIDRα-DBLβ (n = 24/41; 59%), NTS-DBLα-CIDRα-DBLδ (n = 12/41; 29%) and NTS-DBLα-CIDRα-DBLγ (n = 1/41; 2,4%).

**Fig 3:**
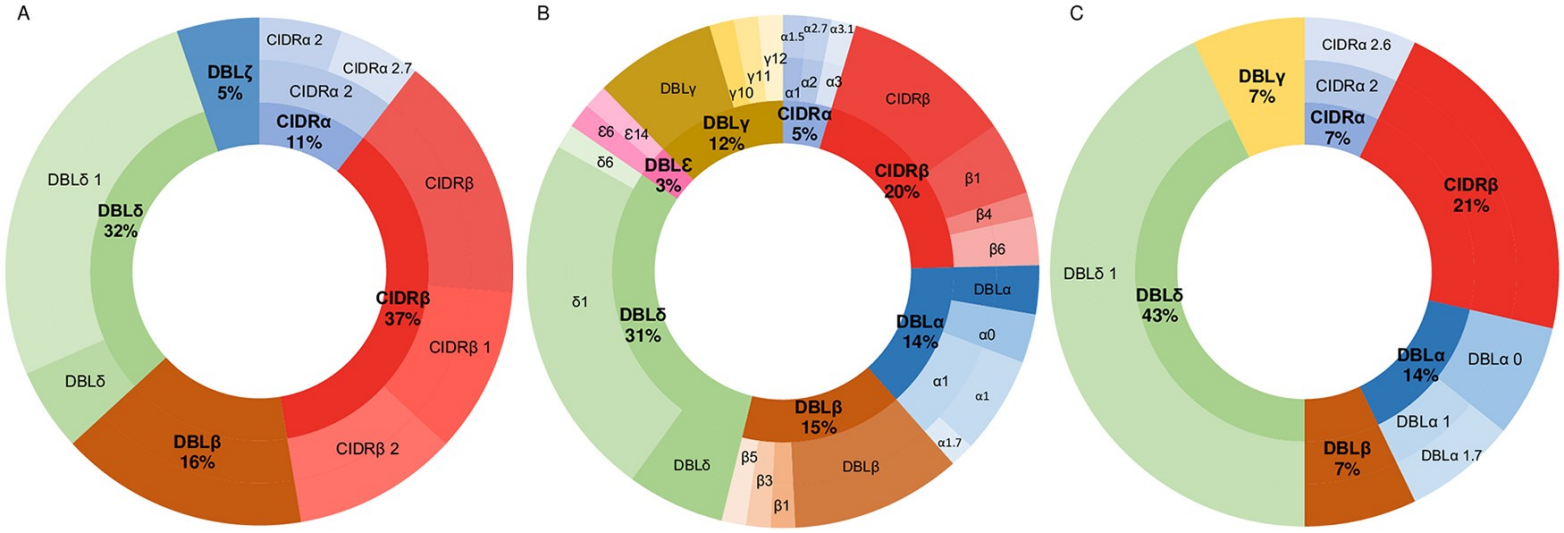
Proportion of peptide corresponding to each PfEMP1 domains and subdomains in association with the clinical outcome. Domains were attributed to each peptide identified in LC-MS/MS. The proportions are displayed for (A) CM patients, (B) SA patients and (C) UM patients.

Considering the difficulties to attribute a given PfEMP1 protein to a sample in this experimental setting, we then focused our analysis on the peptides attributed to PfEMP1 proteins. We identified 147 peptides attributed to PfEMP1, among which 110 were unique peptides (S5 Table). Among these 147 peptides identification, 46 were peptides from the public data repositories, while the remaining ones were specific to the protein sequences identified using WGS. The peptides were distributed as following among the PfEMP1 domains: ATS 14/147 (10%), CIDRα 6/147 (4%), CIDRβ 22/147 (15%), DBLα 11/147 (7%), DBLβ 14/147 (9%), DBLδ 32/147 (22%), DBLε 2/147 (1%), DBLγ 9/147 (6%), DBLζ 1/147 (0.7%), NTS 21/147 (14%) and 15/147 (10%) of the peptides were unattributed. The two majors’ domains identified with the peptides are CIDRβ and DBLδ which are in equivalent proportion in all clinical group (*p =* 0.41 and 0.21 respectively). Regarding the CM samples, no peptide associated to the DBLα was identified (Fig 3).

## Discussion

The evolution of *P*. *falciparum* infection from uncomplicated forms of the disease to cerebral malaria, the most fatal, is a complex phenomenon [44]. There are strong evidences that the PfEMP1 proteins are involved in the disease progression since they allow the parasite to bind to host endothelium [10]. It is believed that a distinct subset of PfEMP1 proteins is involved in severe malaria [23,45], most likely by providing to the parasite the ability to sequester to a given receptor. However, PfEMP1 identification in natural infection remained challenging, due to the large size of PfEMP1 and their high sequences diversity. Recently, Jespersen et *al* [23] provided a new insight towards *var* genes sequences expression analysis in patient’s sample using transcript reconstruction after DBLα barcoding. They confirmed the preferential expression of CIDRα associated with EPCR binding in severe malaria patients. In addition, Tonkin Hill et *al* performed a *de novo* reconstruction of *var* genes from patient’s isolates [28].

We used a mass spectrometry–based proteomic approach to analyse the *P*. *falciparum* proteome in the context of severe malaria (SA and CM) compared to UM. We aimed to accurately identify, at the protein level, the PfEMP1 sequence variants associated with diseases severity. To this end, we initiated a “proteogenomic” study of field samples (Fig 1).

Using reconstructed *var* genes obtained by WGS, we were able to identify the transcript expressed for each isolate among the one from the cognate genome. In addition, we performed a differential expression analysis of the *var* domains. We demonstrated that the domains DBLα1.7/2 and DBLβ12 are a signature of the CM sample. These domains are part of the DC8 and DC13, which are described as involved in the pathogenesis of cerebral malaria in patients from several endemic area [11,20,25,36]. The convergence of our results with the published results in the literature using targeted methods enforce the association of DBLα1.7/2 and DBLβ12 expression and cerebral malaria. We also demonstrated that the *var* expression pattern of the SA patients was distinct from the CM patients, in accordance with the specific sequestration pattern of *P*. *falciparum* in CM pathogenesis.

At the protein level, we were able to identify peptides associated with PfEMP1. As anticipated, most of the identified PfEMP1 came from the newly added sequences to the database (10/57 were known sequences), confirming the validity of our approach considering the high variability of PfEMP1 proteins.

Using peptides fractionation, we identified more proteins than previously published studies [12,46], with higher sequence coverage. We identified a set of 57 PfEMP1 in the studied samples and investigated the structure of theses sequences. Our finding revealed that the two main domain organisations were NTS-DBLα-CIDRα-DBLβ and NTS-DBLα-CIDRα-DBLδ. The high proportion of NTS-DBLα-CIDRα-DBLβ in our identified PfEMP1 proteins compared to genomic sequences within the same sample pool reflects the preferential expression of the PfEMP1 containing this domain association. The CIDRα-DBLβ tandem is associated with the potential “double binding” PfEMP1 [19,24], targeting both ICAM-1 (through DBLβ [19]) and EPCR (through CIDRα [22]) human endothelial receptors. Nevertheless, the highly recombinogenic nature of *var* genes means that the presence of a partial *var* sequence in a *var* gene from one isolate does not mean that if the sequence is present in another isolate that it is present in the same gene. Thus inferring the presence of entire PfEMP1s or domains for which peptides have not been directly obtained must be regarded with caution, with the exception of the atypically conserved var2csa, var1 and var3.

Focusing on the identified peptides, we were able to identify peptides as a signature of a PfEMP1 specific domain. Even though the peptide length might seem short, this is equivalent to the length of the PCR products used in the conventional qPCR approaches to assess specific domain expression in field samples [11,21].

In conclusion, we identified PfEMP1 proteins expressed by parasite in patients presenting several forms of malaria. This is one of the first proteomic report of full PfEMP1 protein direct identification and is providing insight towards malaria pathogenesis understanding. The high proportion of CIDRα among the identified sequences enforce the idea that iE sequestration occurs either through CD36 binding, or EPCR binding, pending of clinical presentation [22,47]. We also preferentially identified PfEMP1 protein harbouring DBLβ, among which 20% (6/30 identified DBLβ) displayed the binding pattern for ICAM-1. In addition, the proportion of peptides corresponding to DBLβ was higher in the severe malaria patients compared to the uncomplicated malaria patients. These strengthen the hypothesis that DBLβ is involved in the disease development, as demonstrated with antibodies against DBLβ in Tanzania [48] and Papua New Guinea [49]. However, the technical limitation of bottom-up approach in LC-MS/MS does not allow for an optimal sequence coverage for precise PfEMP1 variants identification.

Both RNAseq and LC-MS/MS analysis showed that *var* and PfEMP1 involved in CM and SA are distinct. This enforce the necessity to study well characterized clinical group. In addition, severe anaemia is a common complication of *P*. *falciparum* infection in endemic areas [50]. The dedicated *P*. *falciparum var* and PfEMP1 associated phenotype should be further investigated. However, severe anaemia associated malaria is multi-factorial and the clinical outcome might not be solely related to a dedicated *var/*PfEMP1 subtype.

Our study opens opportunities to identify PfEMP1 variants and later implement these newly identified sequences in PfEMP1 based vaccine development strategies [51,52].

Further studies should include patients from various *P*. *falciparum* endemic areas to better represent PfEMP1 associated within *P*. *falciparum* disease in general and specifically to severe malaria.

## Supporting information

S1 Table List of the ENA accession number for the WGS data file. (DOCX)

S2 Table List of the *var* transcripts identified using RNAseq for each sequenced sample with the corresponding RPKM values. For each identified transcript, the domain combination obtained using phylogenic analysis is displayed. The signs + and ++ represents respectively the subdomains of DC8 and DC13.
(XLSX)

S3 Table Sequences of the PfEMP1 proteins identified in LC-MS/MS. (FASTA)

S4 Table Structure of the PfEMP1 proteins identified in LC-MS/MS. (XLSX)

S5 Table List of the peptides identified per sample. The PEP score reflects the probability of true identification of a given peptide. Domain type has been attributed as described in the methods section.
(XLSX)
