# Supplementary material for: From genomic to LC-MS/MS evidence: Analysis of PfEMP1 in Benin malaria cases
Source: PLoS One. 2019 Jun 28;14(6):e0218012. doi: 10.1371/journal.pone.0218012 (PMC6599223; doi:10.1371/journal.pone.0218012)
Supplement: S1 Table — (DOCX) [file pone.0218012.s001.docx]

**ENA accession number for WGS sequences**

| ERS1779107 |  | ERS1779020 |  | ERS1778944 |
| --- | --- | --- | --- | --- |
| ERS1779099 |  | ERS1779036 |  | ERS1778959 |
| ERS1779106 |  | ERS1779027 |  | ERS1778951 |
| ERS1779097 |  | ERS1779019 |  | ERS1778943 |
| ERS1779105 |  | ERS1779035 |  | ERS1778958 |
| ERS1779096 |  | ERS1779026 |  | ERS1778950 |
| ERS1779104 |  | ERS1779018 |  | ERS1778942 |
| ERS1779095 |  | ERS1779034 |  | ERS1778957 |
| ERS1779103 |  | ERS1779025 |  | ERS1778949 |
| ERS1779102 |  | ERS1779017 |  | ERS1778941 |
| ERS1779101 |  | ERS1779041 |  | ERS1778956 |
| ERS1779100 |  | ERS1779033 |  | ERS1778948 |
| ERS1779089 |  | ERS1779024 |  | ERS1778940 |
| ERS1779080 |  | ERS1779040 |  | ERS1778939 |
| ERS1779072 |  | ERS1779032 |  | ERS1778930 |
| ERS1779088 |  | ERS1779023 |  | ERS1778922 |
| ERS1779079 |  | ERS1779039 |  | ERS1778938 |
| ERS1779071 |  | ERS1779031 |  | ERS1778929 |
| ERS1779087 |  | ERS1779022 |  | ERS1778921 |
| ERS1779078 |  | ERS1779038 |  | ERS1778936 |
| ERS1779070 |  | ERS1779030 |  | ERS1778928 |
| ERS1779086 |  | ERS1779021 |  | ERS1778920 |
| ERS1779077 |  | ERS1779016 |  | ERS1778935 |
| ERS1779069 |  | ERS1779007 |  | ERS1778927 |
| ERS1779094 |  | ERS1778996 |  | ERS1778919 |
| ERS1779085 |  | ERS1779015 |  | ERS1778934 |
| ERS1779076 |  | ERS1779006 |  | ERS1778926 |
| ERS1779093 |  | ERS1778995 |  | ERS1778918 |
| ERS1779084 |  | ERS1779014 |  | ERS1778933 |
| ERS1779075 |  | ERS1779005 |  | ERS1778925 |
| ERS1779092 |  | ERS1778994 |  | ERS1778916 |
| ERS1779083 |  | ERS1779013 |  | ERS1778932 |
| ERS1779074 |  | ERS1779003 |  | ERS1778924 |
| ERS1779091 |  | ERS1778993 |  | ERS1778915 |
| ERS1779081 |  | ERS1779012 |  | ERS1778931 |
| ERS1779073 |  | ERS1779002 |  | ERS1778923 |
| ERS1779055 |  | ERS1778992 |  | ERS1778914 |
| ERS1779046 |  | ERS1779011 |  | ERS1778884 |
| ERS1779061 |  | ERS1778991 |  | ERS1778876 |
| ERS1779054 |  | ERS1779010 |  | ERS1778866 |
| ERS1779045 |  | ERS1778998 |  | ERS1778883 |
| ERS1779060 |  | ERS1778990 |  | ERS1778875 |
| ERS1779053 |  | ERS1779009 |  | ERS1778865 |
| ERS1779044 |  | ERS1778997 |  | ERS1778882 |
| ERS1779059 |  | ERS1778989 |  | ERS1778873 |
| ERS1779051 |  | ERS1778988 |  | ERS1778864 |
| ERS1779066 |  | ERS1778980 |  | ERS1778881 |
| ERS1779042 |  | ERS1778971 |  | ERS1778872 |
| ERS1779068 |  | ERS1778987 |  | ERS1778863 |
| ERS1779050 |  | ERS1778979 |  | ERS1778880 |
| ERS1779065 |  | ERS1778970 |  | ERS1778870 |
| ERS1779067 |  | ERS1778986 |  | ERS1778862 |
| ERS1779049 |  | ERS1778977 |  | ERS1778879 |
| ERS1779064 |  | ERS1778969 |  | ERS1778869 |
| ERS1779057 |  | ERS1778985 |  | ERS1778861 |
| ERS1779048 |  | ERS1778976 |  | ERS1778878 |
| ERS1779063 |  | ERS1778968 |  | ERS1778868 |
| ERS1779056 |  | ERS1778984 |  | ERS1778860 |
| ERS1779047 |  | ERS1778975 |  | ERS1778877 |
| ERS1779062 |  | ERS1778967 |  | ERS1778867 |
| ERS1779037 |  | ERS1778983 |  | ERS1778859 |
| ERS1779029 |  | ERS1778974 |  | ERS1778913 |
| ERS1778955 |  | ERS1778966 |  | ERS1778905 |
| ERS1778947 |  | ERS1778982 |  | ERS1778894 |
| ERS1778962 |  | ERS1778973 |  | ERS1778912 |
| ERS1778954 |  | ERS1778965 |  | ERS1778904 |
| ERS1778946 |  | ERS1778981 |  | ERS1778892 |
| ERS1778961 |  | ERS1778972 |  | ERS1778911 |
| ERS1778953 |  | ERS1778964 |  | ERS1778903 |
| ERS1778945 |  | ERS1778963 |  | ERS1778891 |
| ERS1778960 |  | ERS1778978 |  | ERS1778910 |
| ERS1778887 |  | ERS1779004 |  | ERS1778901 |
| ERS1778906 |  | ERS1778999 |  | ERS1778890 |
| ERS1778896 |  | ERS1779008 |  | ERS1778909 |
| ERS1778886 |  | ERS1779028 |  | ERS1778899 |
| ERS1778898 |  | ERS1778907 |  | ERS1778889 |
| ERS1778888 |  | ERS1778897 |  | ERS1778908 |
